# Tyrosine Hydroxylase Phosphorylation in Catecholaminergic Brain Regions: A Marker of Activation following Acute Hypotension and Glucoprivation

**DOI:** 10.1371/journal.pone.0050535

**Published:** 2012-11-29

**Authors:** Hanafi A. Damanhuri, Peter G. R. Burke, Lin K. Ong, Larisa Bobrovskaya, Phillip W. Dickson, Peter R. Dunkley, Ann K. Goodchild

**Affiliations:** 1 The Australian School of Advanced Medicine, Macquarie University, North Ryde, New South Wales, Australia; 2 School of Biomedical Sciences and Pharmacy, The University of Newcastle, Callaghan, New South Wales, Australia; 3 School of Pharmacy and Medical Sciences, University of South Australia, Adelaide, South Australia, Australia; 4 Biochemistry Department, Faculty of Medicine, Universiti Kebangsaan Malaysia, Kuala Lumpur, Malaysia; University of California, Los Angeles, United States of America

## Abstract

The expression of c-Fos defines brain regions activated by the stressors hypotension and glucoprivation however, whether this identifies all brain sites involved is unknown. Furthermore, the neurochemicals that delineate these regions, or are utilized in them when responding to these stressors remain undefined. Conscious rats were subjected to hypotension, glucoprivation or vehicle for 30, 60 or 120 min and changes in the phosphorylation of serine residues 19, 31 and 40 in the biosynthetic enzyme, tyrosine hydroxylase (TH), the activity of TH and/or, the expression of c-Fos were determined, in up to ten brain regions simultaneously that contain catecholaminergic cell bodies and/or terminals: A1, A2, caudal C1, rostral C1, A6, A8/9, A10, nucleus accumbens, dorsal striatum and medial prefrontal cortex. Glucoprivation evoked phosphorylation changes in A1, caudal C1, rostral C1 and nucleus accumbens whereas hypotension evoked changes A1, caudal C1, rostral C1, A6, A8/9, A10 and medial prefrontal cortex 30 min post stimulus whereas few changes were evident at 60 min. Although increases in pSer19, indicative of depolarization, were seen in sites where c-Fos was evoked, phosphorylation changes were a sensitive measure of activation in A8/9 and A10 regions that did not express c-Fos and in the prefrontal cortex that contains only catecholaminergic terminals. Specific patterns of serine residue phosphorylation were detected, dependent upon the stimulus and brain region, suggesting activation of distinct signaling cascades. Hypotension evoked a reduction in phosphorylation in A1 suggestive of reduced kinase activity. TH activity was increased, indicating synthesis of TH, in regions where pSer31 alone was increased (prefrontal cortex) or in conjunction with pSer40 (caudal C1). Thus, changes in phosphorylation of serine residues in TH provide a highly sensitive measure of activity, cellular signaling and catecholamine utilization in catecholaminergic brain regions, in the short term, in response to hypotension and glucoprivation.

## Introduction

Hypotension and glucoprivation are two physical stressors. Hypotension unloads baroreceptors, which leads to the activation or inhibition of a number of brain sites in an attempt to restore arterial pressure (AP). Similarly, glucoprivation, which results in low effective plasma glucose concentration, leads to the activation of centrally mediated counter regulatory events in order to restore plasma glucose levels. The brain sites activated by these stimuli have been demonstrated using the expression of c-Fos to mark activated neurons. With respect to hypotension limited brain sites including the nucleus of the solitary tract (NTS), ventrolateral medulla (VLM), A5 cell group, A6 cell group (not in rat), periaquaductal grey (PAG), central amygdaloid nucleus (CeA), supraoptic nucleus (SON), lateral hypothalamic area (LHA), paraventricular nucleus (PVN), and bed nucleus of the stria terminalis (BNST) are activated [1]–[3], whereas with glucoprivation the NTS, VLM, A6, PAG, CeA, LHA, dorosmedial hypothalamus (DMH), PVN, ventral palladis (VP) and nucleus accumbens (NAc) are activated [4]–[6]. Although this technique has proven extremely valuable, little is known about the neurotransmitters/neurochemistry involved, the brain sites that may be inhibited, whether c-Fos is turned on in all sites that are activated or the time course of effect.

The approach taken here was to determine the involvement of the catecholamines (dopamine, noradrenaline and adrenaline) within the brain, in response to hypotensive and glucoprivic stress. Multiple catecholamine cell groups are found within the brain, each capable of synthesizing one of the three catecholamines. The end product in each cell group depends upon the synthetic enzymes present. Tyrosine hydroxylase (TH) is the initial and rate limiting enzyme for all catecholamine biosynthesis. We will take advantage of the short term regulation of TH via phosphorylation of three serine residues at the N-terminal domain that can directly or indirectly result in increased activity of the enzyme [7]–[8]. This process is well understood under *in vitro* conditions [8] and evidence of TH phosphorylation in brain regions has been demonstrated using ^32^P incorporation [9] and western blotting, using phosphospecific TH antibodies [10]–[15]. It should be noted that Ser19, Ser31 and Ser40 are the only phosphorylation sites recognized to directly or indirectly change the activity of TH or be phosphorylated under *in vivo* or *in situ* conditions [8]. However the effects of hypotension or glucoprivation have not been investigated in the brain using such an approach.

Specific kinases are responsible for the phosphorylation of the three serine residues on the N terminus of TH [8], [16]. For example, calcium/calmodulin dependent protein kinase II (CaMKII) is predominantly responsible for the phosphorylation of Ser19, mitogen-activated protein kinase (MAPK) or cyclin-dependent kinase 5 (CDK5) are the only kinases known to phosphorylate Ser31, while multiple protein kinases can phosphorylate Ser40. Thus some evidence as to the intracellular signaling pathways activated by hypotensive and glucoprivic stressors can also be determined by investigating the serine residues on TH that are phosphorylated in response to these stressors.

In the adrenal medulla, another site of catecholamine synthesis, catecholamine release induces TH phosphorylation and enzyme activity increases in order to maintain the catecholamine stores [7], [17]–[18]. Thus any increase in TH activity indicates that the catecholamine has been released, or metabolized, or reuptake has been blocked.

Catecholamines could be utilized in the central response evoked by glucoprivation or hypotension. Following hypotension or glucoprivation the medullary C1 cell group expresses c-Fos [2], [6]. Similarly selective lesioning of catecholaminergic cell groups, using anti-DBH saporin, shows that C1 neurons are required for the full expression of the sympathetic baroreflex [19] and the sympathoadrenal response to glucoprivation [20]. However these studies do not indicate whether or not adrenaline is utilized by these neurons. On the other hand, direct measurement of catecholamine release in response to hypotension has been performed, using *in vivo* dialysis, and this powerful technique further provides evidence of the time course of response although only single or at most two central sites can be explored simultaneously. Catecholamines are released in prefrontal cortex and A6 following hypotension [21], whereas glucoprivation or hypoglycemia evoke noradrenaline release in select hypothalamic areas although the latency to response is increased [22]–[23]. Curiously common links between hypotension evoked c-Fos activated brain sites and catecholamine release are limited to A6 further emphasizing the lack of knowledge regarding the involvement of catecholamines in response to stressors such as hypotension and glucoprivation.

Thus, the major aims of this study were to

1. Determine, at ten brain sites simultaneously, the response to acute hypotension or glucoprivation by identifying the changes in phosphorylation at Ser19, Ser31 and Ser40 in TH in order to determine the involvement of catecholamines and catecholaminergic structures in mediating the counter regulatory responses.
2. Compare and contrast the brain sites and phosphorylation patterns activated/inhibited by hypotension or glucoprivation at two time points 30 and 60 min post stimulus.

Using the data generated in combination with previous literature we can also deduce information related to:

1. The intracellular signaling processes that result in phosphorylation of TH and/or release of catecholamine.
2. The neural substrate (terminal field or soma) responsible for the changes in TH phosphorylation evoked.

We have demonstrated that changes in phosphorylation of TH were evoked in multiple and distinct brain regions dependent upon the stimulus. The approach used here is more sensitive than other methods for detecting activation as sites not previously described are activated, the effects can be detected earlier, a time course of response can be determined, the effect is neurochemically selective, activation of terminal fields alone can be identified and bidirectional changes in phosphorylation can be detected. In addition, the utilization of catecholamines in some brain regions mediating baroreceptor reflex function and the response to glucoprivation is indicated.

## Results

### The Effect of Hydralazine (HDZ) or 2- Deoxy-D-glucose (2DG) on Arterial Pressure (AP), Heart Rate (HR), Blood Glucose and Plasma Corticosterone

**Figure 1:**
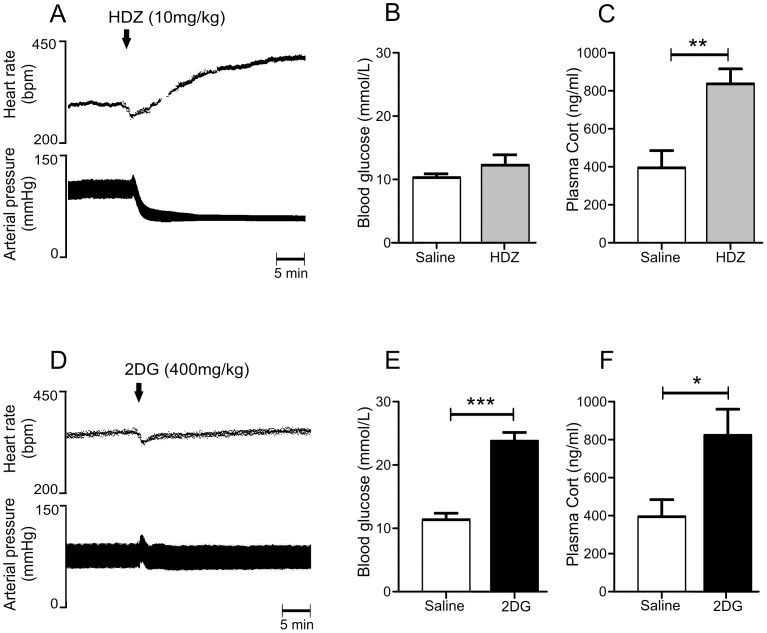
Physiological effects evoked by hydralazine (HDZ; A–C) or 2-deoxyglucose (2DG: D–F). A) HDZ (10 mg/kg i.v.) evokes a profound fall in arterial pressure. HDZ did not alter blood glucose (B) but significantly increased plasma corticosterone (Cort, C). In contrast 2DG (400 mg/kg i.v.) did not alter arterial pressure or heart rate but evoked significant increases in both blood glucose (E) and plasma corticosterone (Cort, F).

The administration of hydralazine (HDZ, 10 mg/kg i.v.), a potent vasodilator, caused a persistent hypotension, which evoked a baroreflex mediated increase in HR (Fig. 1A) as described previously [3], [24]. Thirty min after HDZ administration blood glucose levels did not change (Fig. 1B), whereas plasma corticosterone was increased 2.1 fold (p<0.01, Fig. 1C) compared to levels seen after saline administration. In contrast no persistent change was evoked in AP or HR following 2-Deoxy-D-glucose (2DG, 400 mg/kg i.v.) administration, an agent causing glucoprivation (Fig. 1D). Thirty min after the administration of 2DG blood glucose was elevated 2.1 fold (p<0.001, Fig. 1E) and plasma corticosterone was increased 2.1 fold (p<0.05, Fig. 1F) compared with results seen following saline administration.

### The Effects of HDZ or 2DG on TH Phosphorylation in Catecholaminergic Cell Groups in the Brain

**Figure 2:**
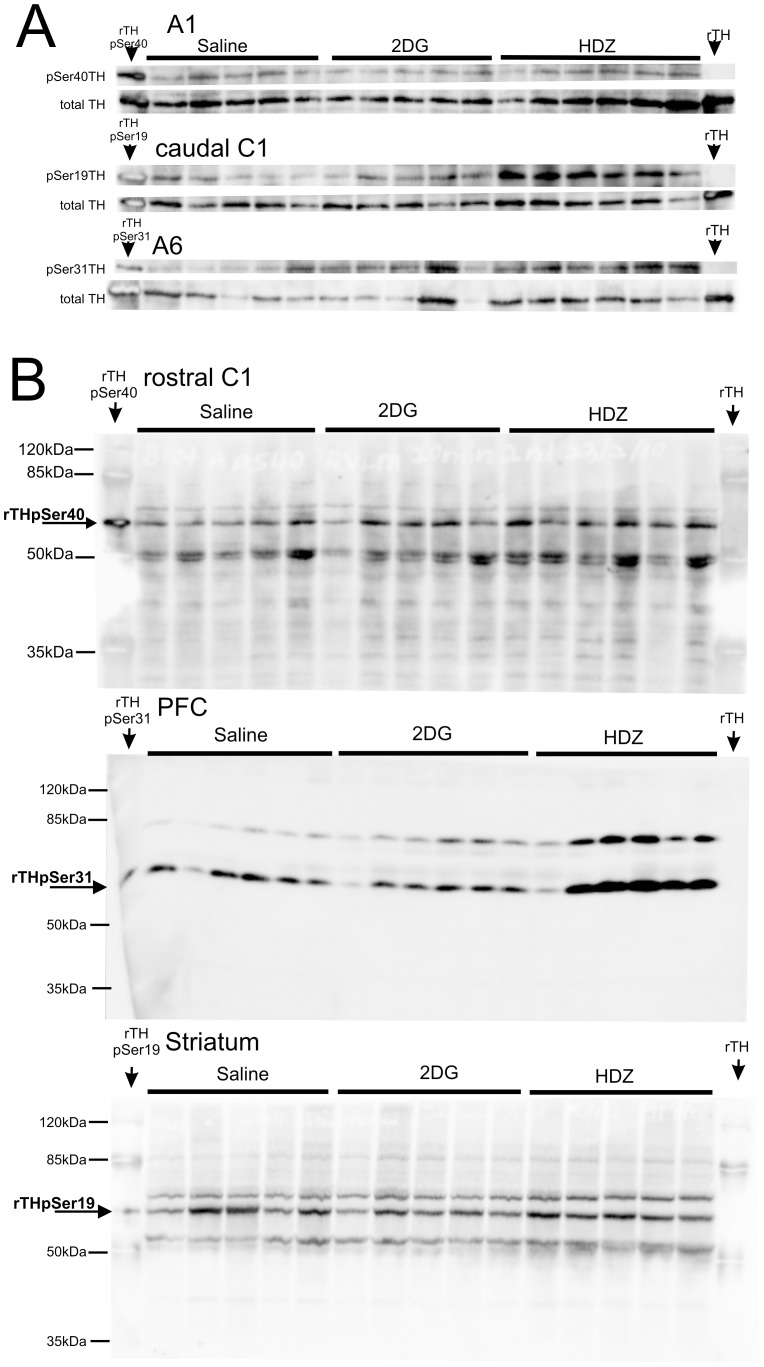
Representative western blots from six brain regions comparing the effects of saline, 2DG and HDZ treatment. A) Western blots from three brain regions: A1, cC1 and A6 showing the results using phospho-specific and TH antibodies and total TH. Recombinant TH, both site specific phosphorylated and non phosphorylated, are shown on each blot and mark the band measured. Please note the order of the stimuli is different to that shown in later figures. Each lane on the blot represents a different animal. B) Full length western blots from three different brain regions: rC1, mPFC and striatum. Recombinant TH, both site specific phosphorylated and non phosphorylated, are shown on each blot and mark the band analysed. Also shown in the lanes containing the recombinant TH are molecular weight markers with sizes indicated.

**Figure 3:**
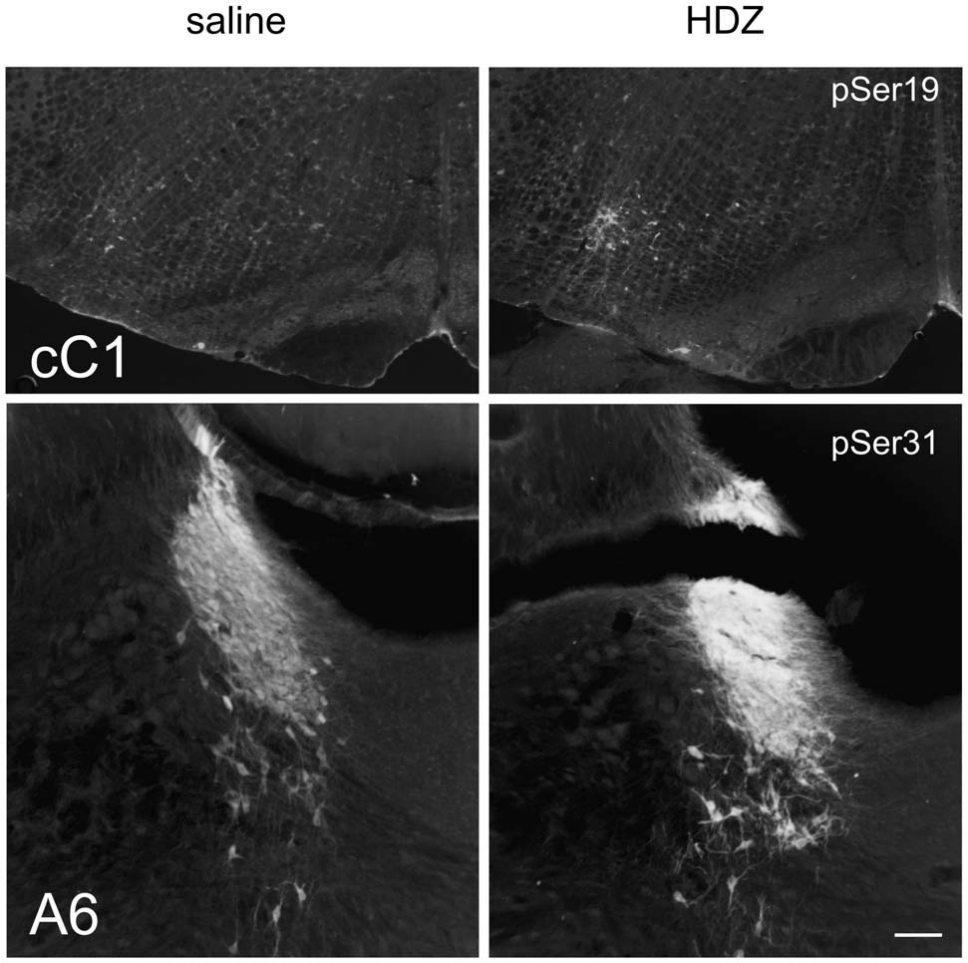
Examples of immunohistochemical labeling using antibodies against pSer19TH and pSer31TH in cC1 and A6 regions following saline or HDZ treatment. These regions were selected as the effects of HDZ at these sites were demonstrated to be robust using Western blot analysis (see Fig. 2). Quantitative analysis was not performed (see text).

Using phospho-specific antibodies, the level of TH phosphorylation at specific serine residues was determined in 10 brain regions, 30 min after HDZ (n = 5–6), 2-DG (n = 5–6) or saline (n = 5–6) administration using western blot analysis. Recombinant rat TH (rTH) specifically phosphorylated to full stoichiometry at Ser40, or Ser31, or Ser19 (see methods) and also non-phosphorylated recombinant rat TH were run on each blot. Figure 2A shows blots from 3 brain regions and also shows the TH protein blot. There was no significant effect of the treatments on TH protein levels in any region investigated (not shown). Figure 2B shows full length blots from 3 different brain regions using phospho-specific specific antibodies run using rTH, as described above, together with molecular weight markers. In addition, images shown in Figure 3 demonstrate the capacity of these antibodies to be used to localise phosphorylated TH immunoreactivity. Although it is very difficult to quantify such data some evidence of increased phosphorylation evoked by hydralazine compared to saline treatment may be seen in the cC1 and A6 regions using pSer19 and pSer31 antibodies (Fig. 3).The results of western blot analysis in subsequent Figures are all expressed as Ser40 phosphorylation/total TH, Ser31 phosphorylation/total TH and Ser19 phosphorylation/total TH for each brain region. In addition select brain regions were also subjected to analysis 60 min after HDZ (n = 5), 2DG (n = 5) or saline (n = 5) administration. The data was then normalized by setting the level of site specific phosphorylation of TH following saline administration to 1 and the data was then compared with that found for the two treatments. The format of subsequent figures are similar. The schematic sections in each were adapted from Paxinos and Watson (2005) the coronal section shown represents approximately the mid rostrocaudal level for each brain region examined. The grey shaded region in each section represents the tissue region analyzed.

### Noradrenergic and Adrenergic Cell Groups

#### The A1 cell region

**Figure 4:**
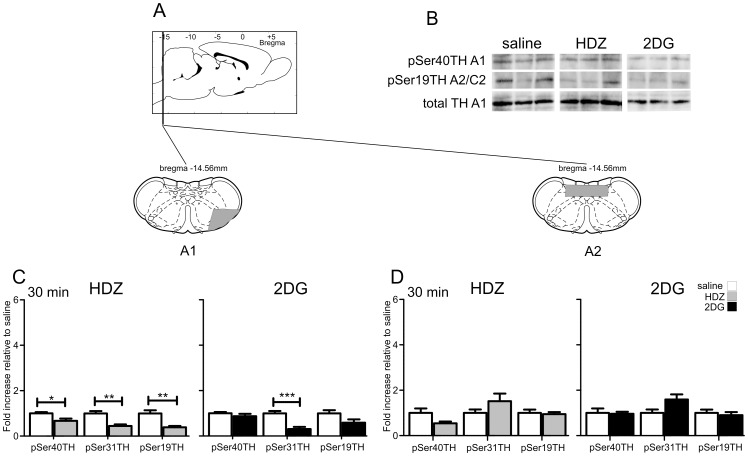
TH phosphorylation evoked by HDZ (10 mg/kg i.p., n = 6) or 2DG (400 **mg/kg i.p., n = 5) compared to saline (i.p. n = 5) in the A1 and A2 regions 30 min after the stimulus.** A) shows schematically the rostrocaudal level and coronal section at the midpoint of tissue extracted for each region examined. The grey shaded regions depict the areas analyzed. B) Examples of western blots that were used for analysis where each lane represents a single animal. Site-specific TH phosphorylation was expressed as the ratio of TH phosphorylation at Ser19, Ser31 or Ser40 to total TH protein, for each treatment group. The data was normalized by setting the ratio following saline administration to one and the data compared with that for the two treatments. C) In the A1 region HDZ caused significant decreases in pSer40, pSer31 and pSer19 whereas 2DG evoked a significant decrease in pSer31 only. D) Neither HDZ nor 2DG evoked any changes in TH phosphorylation in the A2 region. * P<0.05, ** P<0.01, *** P<0.001.

Figure 4A shows the two most caudal catecholaminergic cell groups analyzed: the A1 cell group and the A2 cell group. Representative western blots are shown in Figure 2 and 4B. In the A1 cell group, 30 min following HDZ administration, decreases in TH phosphorylation were seen at Ser40 (−1.5 fold, p<0.05, Fig. 4C), Ser31 (−2.2 fold, p<0.01, Fig. 4C) and Ser19 (−2.6 fold, p<0.01, Fig. 4C), whereas 30 min after 2DG administration a decrease in TH phosphorylation was seen at Ser31 (−3.3 fold, p<0.001, Fig. 4C) with no changes evident at Ser40 and Ser19.

#### The A2 cell region

In the A2 cell region no changes in phosphorylation at any serine sites were seen 30 min after either stimulus (Fig. 4D).

#### The C1 cell region

**Figure 5:**
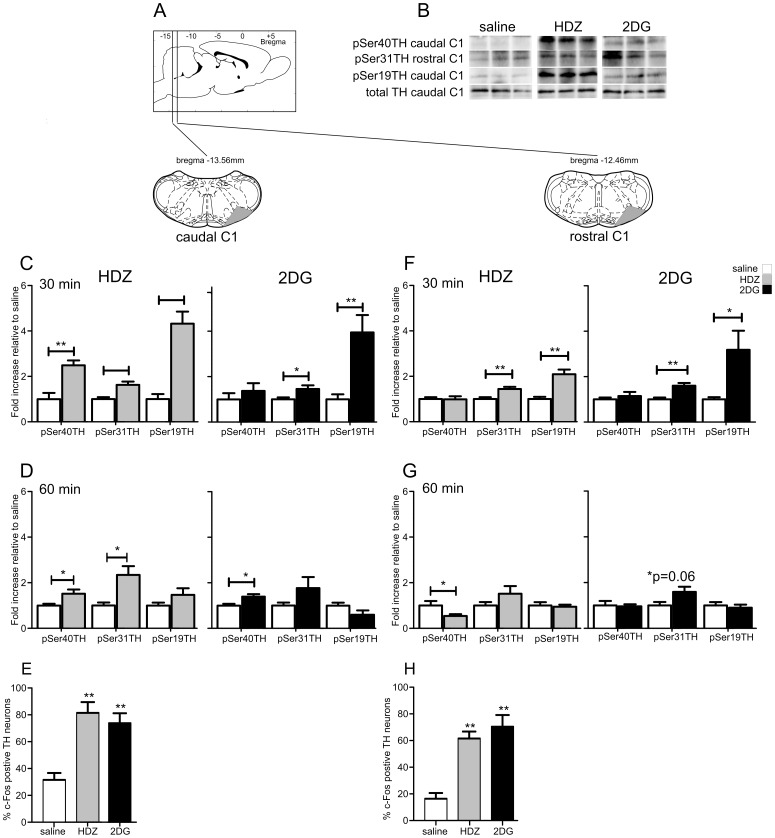
TH phosphorylation evoked by HDZ (10 mg/kg i.p., n = 6) or 2DG (400 **mg/kg i.p., n = 5) compared to saline (i.p. n = 5) in the caudal C1 (C–E) and rostral C1 (F–H) regions.** A) shows schematically the rostrocaudal level and coronal section at the midpoint of tissue extracted for each region examined. The grey shaded regions depict the areas extracted. B) Examples of western blots used for analysis where each lane represents a single animal. Details of analysis are described in the legend for Figure 2. C) In caudal C1 30 min after HDZ significant increases in pSer40, pSer31 and pSer19 were evoked whereas 30 min after 2DG significant increases were seen in pSer31 and pSer19. D) 60 min after HDZ the increases in pSer40 and pSer31 remain whereas an increase in pSer40 emerged 60 min following 2DG. E) Although some c-Fos-ir nuclei were evident in the caudal C1 cells 2 hours after saline most caudal C1 cells expressed c-Fos-ir 2 hours following administration of HDZ or 2DG. F) In the rostral C1 region 30 min following either HDZ or 2DG significant increases in pSer31 and pSer19 were evoked. G) 60 min after HDZ a significant decrease was seen in pSer40 whereas all other phosphorylations had returned to baseline. H) Although a few rostral C1 neurons expressed c-Fos-ir 120 min following saline injection (n = 3) both HDZ (n = 3) and 2DG (n = 3) evoked significant increases in the number of c-Fos-ir rostral C1 neurons. * P<0.05, ** P<0.01, *** P<0.001.

The C1 cell group was analyzed in two parts, the caudal C1 and rostral C1 cell regions and effects were determined at both 30 and 60 min following administration of HDZ, 2DG or saline (Fig. 5A). Representative western blots are shown in Figure 5B and for cC1 also in Figure 2.

**Figure 6:**
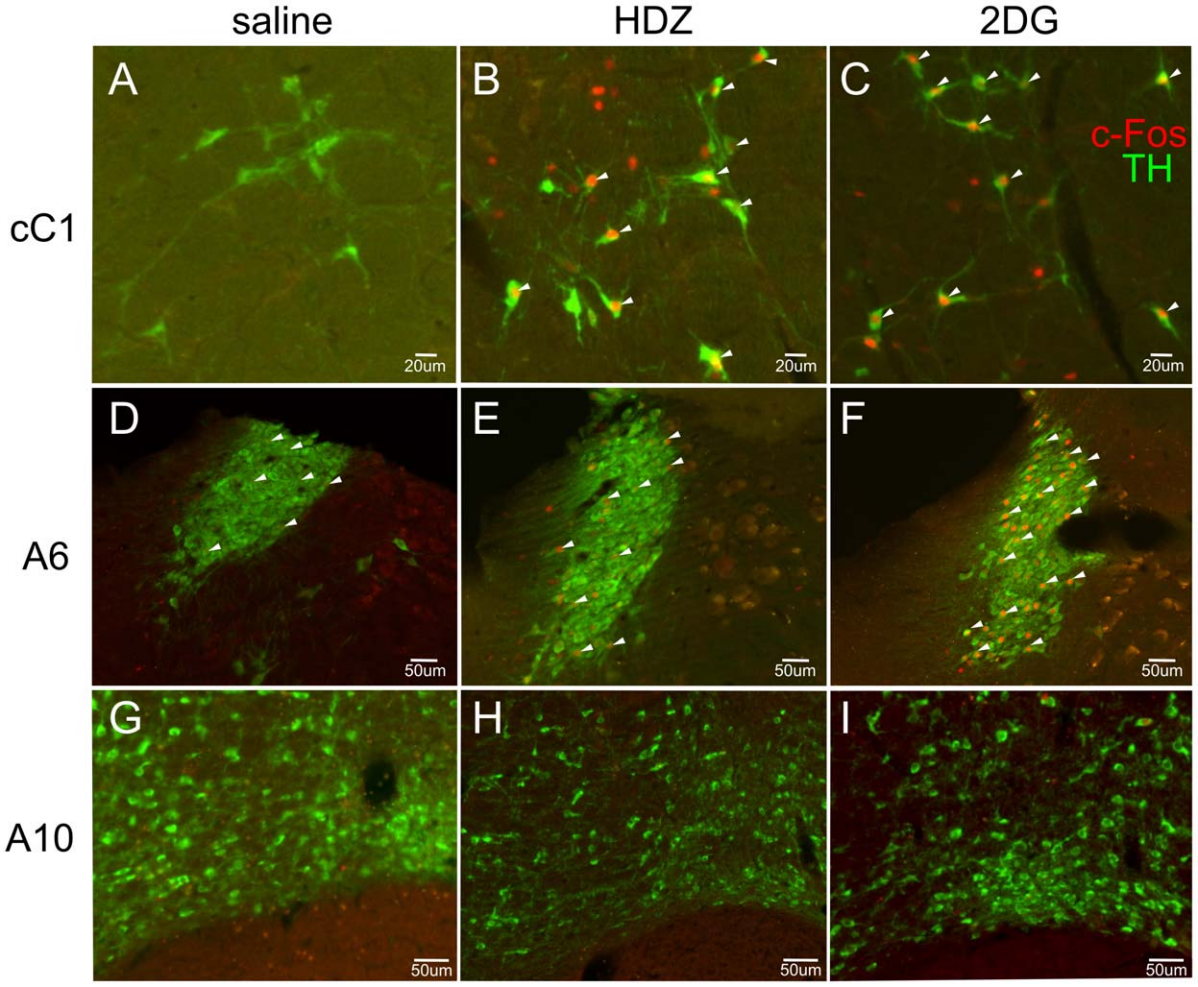
c-Fos-ir tyrosine hydroxylase (TH) containing neurons evoked 120 min following injection (i.p.) of saline (A,D,G), HDZ (B,E,H) or 2DG (C,F,I) in the caudal C1 (A–C), the A6 (D–F) and A10 (G–I) cell groups. TH-ir neurons are green and c-Fos-ir nuclei are red. Few c-Fos-ir neurons are present after saline in all cell groups. c-Fos-ir nuclei are present in many caudal C1 neurons following either HDZ or 2DG. A small number of c-Fos-ir nuclei are present in A6 following HDZ whereas many are present following 2DG. Neither stimulus evoked any c-Fos-ir in the A10 cell group.

In the caudal C1 region, 30 min following HDZ administration, increases in TH phosphorylation were evoked at Ser40 (2.5 fold, p<0.01, Fig. 5C), Ser31 (1.6 fold, p<0.01, Fig. 5C) and Ser19 (4.3 fold, p<0.001, Fig. 5C). However 60 min after HDZ a different pattern of phosphorylation was evident where phosphorylation at Ser19 had returned to basal levels, yet TH phosphorylation was still marked at Ser40 (1.5 fold, p<0.05, Fig. 5D) and increasing at Ser31 (2.3 fold, p<0.05, Fig. 5D). Thirty min following 2DG administration large increases in TH phosphorylation were elicited at Ser31 (1.5 fold, p<0.05, Fig. 5C) and at Ser19 (3.9 fold increase, p<0.01, Fig. 5C). However 60 min after 2DG administration phosphorylation of Ser31 and Ser19 was no longer evident, while phosphorylation at Ser40 (1.4 fold, p<0.05, Fig. 5D) now reached significance mainly due to reduced variance. In order to determine whether or not TH containing neurons (as opposed to terminals) could have contributed to the site specific phosphorylation the level of neuronal activation was determined by the expression of c-Fos immunoreactivity (ir) evoked following the administration of HDZ, 2DG or saline (Fig. 6A–C). c-Fos-ir in the caudal C1 cells was seen following saline administration in 32±5% of TH neurons (n = 3, 32±4 TH neurons counted) whereas 2.6 and 2.3 fold more labelling was seen following HDZ or 2DG, respectively (HDZ 81±8%, n = 3, 35±3 TH neurons counted; 2DG 74±7%, n = 3, 41±7 TH neurons counted Fig. 5E).

In the rostral C1 region, 30 min after HDZ administration TH phosphorylation was increased at Ser31 (1.4 fold, p<0.01, Fig. 5F) and at Ser19 (2.1 fold, p<0.01, Fig. 5F) whereas at 60 min after the stimulus changes were no longer evident at Ser31 and Ser19 and a decrease in phosphorylation was now seen at Ser40 (−1.8 fold, p<0.05, Fig. 5G). A similar pattern of phosphorylation was seen 30 min following 2DG as that seen following HDZ with a 1.6 fold increase at Ser31 (p<0.01, Fig. 5F) and a 3.2 fold increase at Ser19 (p<0.05, Fig. 5F). TH phosphorylation was back to saline levels 60 min after 2DG. In catecholaminergic cell activation only some c-Fos-ir neurons were seen following saline (16±4%, n = 3, 45±5 TH neurons counted, Fig. 5H) with significantly more seen after HDZ (3.8 fold, p<0.01; 62±5%, n = 3, 44±2 TH neurons counted) or 2DG (4.3 fold, p<0.01, 70±9%, n = 3, 46±8 TH neurons counted).

#### The A6 cell region

**Figure 7:**
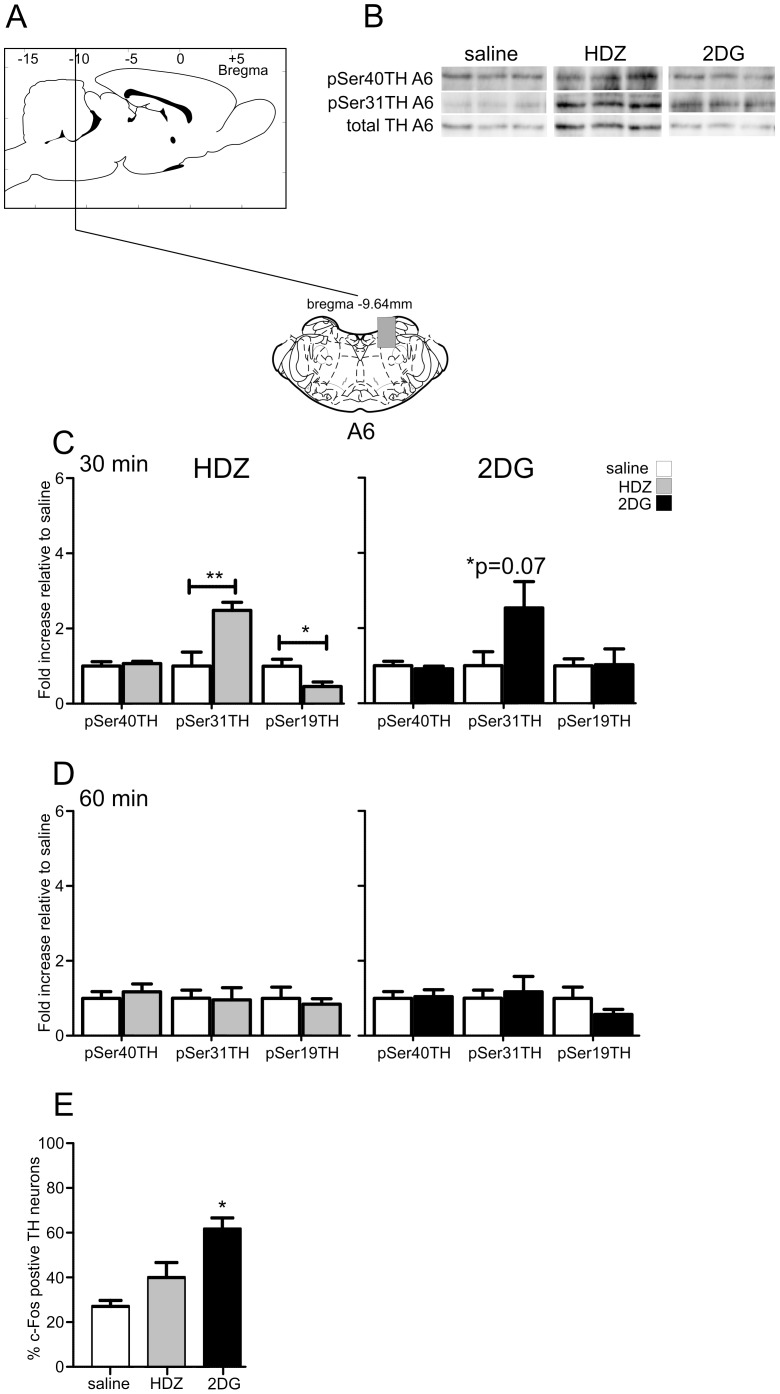
TH phosphorylation evoked by HDZ (10 mg/kg i.p., n = 6) or 2DG (400 **mg/kg i.p., n = 5) compared to saline (i.p. n = 5) in the A6 region.** A) shows schematically the rostrocaudal level and coronal section at the midpoint of tissue extracted for each region examined. The grey shaded regions depict the areas extracted. B) Examples of western blots used for analysis where each lane represents a single animal. Details of analysis are described in the legend for Figure 2. C) In A6 30 min after HDZ significant increases in pSer31 but decreases in pSer19 were evoked whereas 30 min after 2DG an increase was seen in pSer31 although this did not reach significance. D) In A6 60 min after both stimuli all phosphorylation changes seen had returned to baseline. E) Although some c-Fos-ir nuclei were evident in A6 2 hours after saline (n = 3), HDZ (n = 3) evoked no significant effect whereas 2DG (n = 3) caused a significant number of A6 cells to express c-Fos-ir. * P<0.05, ** P<0.01, *** P<0.001.

The A6 cell region was analyzed (Fig. 7A) at 30 and 60 min after the stimuli. Representative western blots are shown in Figure 2 and 7B. TH phosphorylation was evoked 30 min following HDZ at Ser31 (2.5 fold, p<0.01, Fig. 7C) and a decrease in phosphorylation was elicited at Ser19 (−2.2 fold, p<0.05, Fig. 7C) and these changes had returned to baseline 60 min after treatment. 2DG administered for 30 or 60 min showed no significant differences in phosphorylation at any serine sites (Fig. 7D). Some c-Fos–ir neurons were present following saline administration (27±3%, n = 2, 182 and 164 TH neurons counted, Fig. 6D & 7E) with similar numbers of neurons activated following HDZ (40±7%, n = 2, 90 and 193 TH neurons counted, Fig. 6E & 7E), whereas 2DG significantly increased the number of c-Fos-ir cells (2.3 fold, p<0.05, 62±5%, n = 2, 212 and 60 TH neurons counted; saline Fig. 6F & 7E).

### Midbrain Dopaminergic Cell Groups

**Figure 8:**
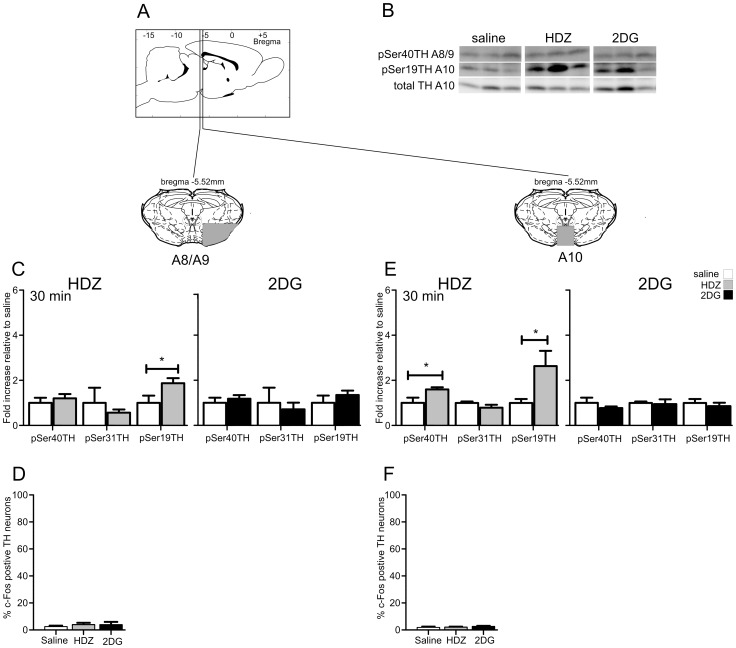
TH phosphorylation evoked by HDZ (10 mg/kg i.p., n = 6) or 2DG (400 **mg/kg i.p., n = 6) compared to saline (i.p. n = 6) in the A8/9 (C,D) and A10 regions (E,F).** A) shows schematically the rostrocaudal level and coronal section at the midpoint of tissue extracted for each region examined. The grey shaded regions depict the areas extracted. B) Examples of western blots used for analysis where each lane represents a single animal. Details of analysis are described in the legend for Figure 2. C) In A8/9 30 min after HDZ significant increases were seen in pSer19 whereas 30 min after 2DG no changes in phosphorylation were evoked. D) Very few c-Fos-ir nuclei were evident in A8/9 2 hours after saline, HDZ or 2DG. E) In A10 30 min after HDZ significant increases were evoked in pSer40 and pSer19 whereas 30 min after 2DG no changes in phosphorylation were evoked. F) Very few c-Fos-ir nuclei were seen in A10 120 min following saline (n = 3), HDZ (n = 3) or 2DG (n = 3). * P<0.05, ** P<0.01, *** P<0.001.

The A8/9 and A10 cell regions were extracted from the same brain sections (Fig. 8A). Representative western blots are shown in Figure 8B.

#### The A8/A9 cell region

In the A8/A9 cell region, 30 min after HDZ administration TH phosphorylation was elevated at Ser19 (1.9 fold, p<0.05, Fig. 8D) with no differences seen at Ser40 and Ser31, whereas no changes in TH phosphorylation were seen 30 min after 2DG (Fig. 8C). Interestingly, the limited number of neurons that were found to be c-Fos-ir rarely contained TH-ir and few double labeled cells were seen following administration of HDZ (5±2%, n = 3, 418±23 TH neurons counted), 2DG (5±3%, n = 3, 402±84 TH neurons counted) or saline (3±1%, n = 3, 390±1 TH neurons counted, Fig. 8D).

#### The A10 cell region

HDZ evoked a greater response in the A10 cell region. Thirty min after HDZ administration, TH was phosphorylated at Ser40 (1.6 fold, p<0.05, Fig. 8E) and Ser19 (2.6 fold, p<0.05, Fig. 8E), whilst 2DG failed to evoke any change in TH phosphorylation (Fig. 8E). Again few c-Fos-ir neurons were present no matter what the treatment and most were not TH-ir (Fig. 6G-I). Similar small numbers of double labeled neurons were seen following all treatments (saline 2.4±1.5%, n = 3, 188±14 TH neurons counted; HDZ 2.8±0.7%, n = 3, 246±23 TH neurons counted; 2DG 3.8±0.7%, n = 3, 251±57 TH neurons counted; Fig. 6G–I, 8F).

### The Effects of HDZ and 2DG on Tyrosine Hydroxylase Containing Terminal Fields in the Brain

**Figure 9:**
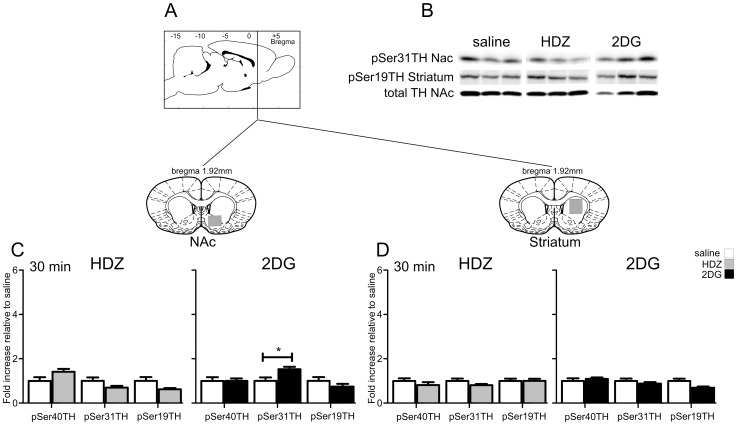
TH phosphorylation evoked by HDZ (10 mg/kg i.p., n = 5) or 2DG (400 **mg/kg i.p., n = 5) compared to saline (i.p. n = 5) in the nucleus accumbens (C) and the dorsal striatum (D).** A) shows schematically the rostrocaudal level and coronal section at the midpoint of tissue extracted for each region examined. The grey shaded regions depict the areas extracted. B) Examples of western blots used for analysis where each lane represents a single animal. Details of analysis are described in the legend for Figure 2. C) In the nucleus accumbens 30 min after HDZ no significant phosphorylation was seen whereas following 2DG an increase in pSer31was evident. D) In the dorsal striatum 30 min after either HDZ or 2DG no changes in phosphorylation were evoked. * P<0.05, ** P<0.01, *** P<0.001.

**Figure 10:**
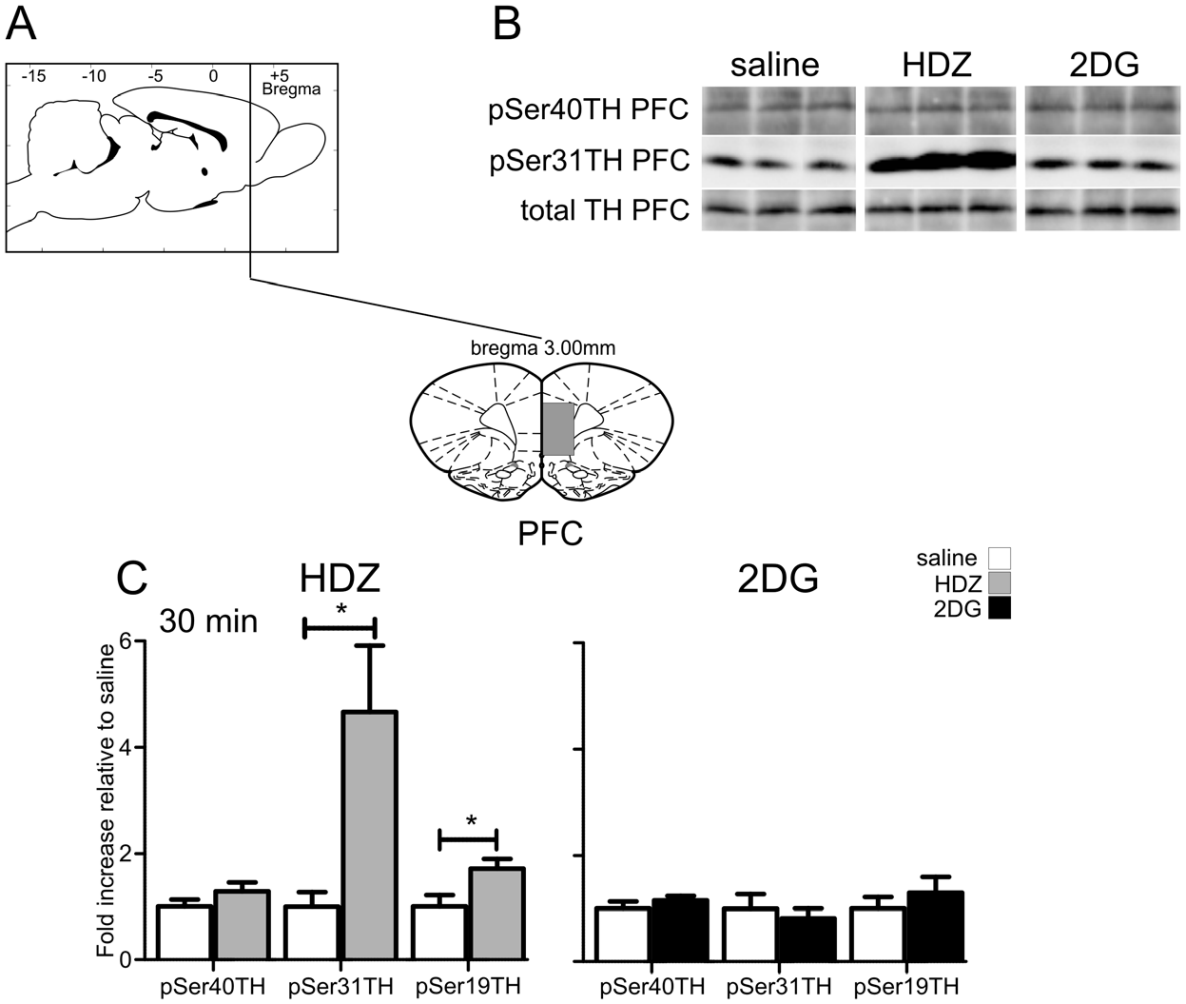
TH phosphorylation evoked by HDZ (10 mg/kg i.p., n = 6) or 2DG (400 **mg/kg i.p., n = 5) compared to saline (i.p. n = 6) in the medial prefrontal cortex (mPFC).** A) shows schematically the rostrocaudal level and coronal section at the midpoint of tissue extracted for each region examined. The grey shaded regions depict the areas extracted. B) Examples of western blots used for analysis where each lane represents a single animal. Details of analysis are described in the legend for Figure 2. C) In the mPFC 30 min after HDZ a significant increase in pSer31 and pSer19 were evident whereas 30 min after 2DG no changes in phosphorylation were evoked. * P<0.05, ** P<0.01, *** P<0.001.

In contrast to regions described above, the nucleus accumbens, dorsal striatum (Fig. 9A) and medial prefrontal cortex (Fig. 10A) do not contain TH expressing cell bodies but are extensively innervated by TH-ir terminal fields [25]. Representative western blots are shown in Figures 9B and 10B.

#### Nucleus accumbens and dorsal striatum

In the nucleus accumbens (NAc), 30 min following HDZ administration TH phosphorylation was not changed at any serine residue (Fig. 8C), whereas phosphorylation at Ser31 was found to be significantly elevated 30 min after 2DG (1.5 fold, p<0.05, Fig. 9C). No changes in TH phosphorylation were seen in the dorsal striatum (Striatum) 30 min after HDZ or 2DG (Fig. 9D).

#### The medial prefrontal cortex

In the medial prefrontal cortex, 30 min after HDZ treatment TH phosphorylation was increased at Ser31 (4.7 fold, p<0.05, Fig. 9C) and at Ser19 (1.7 fold, p<0.05, Fig. 10C). In contrast 2DG administration had no effect on TH phosphorylation in the medial prefrontal cortex (Fig. 10C).

**Figure 11:**
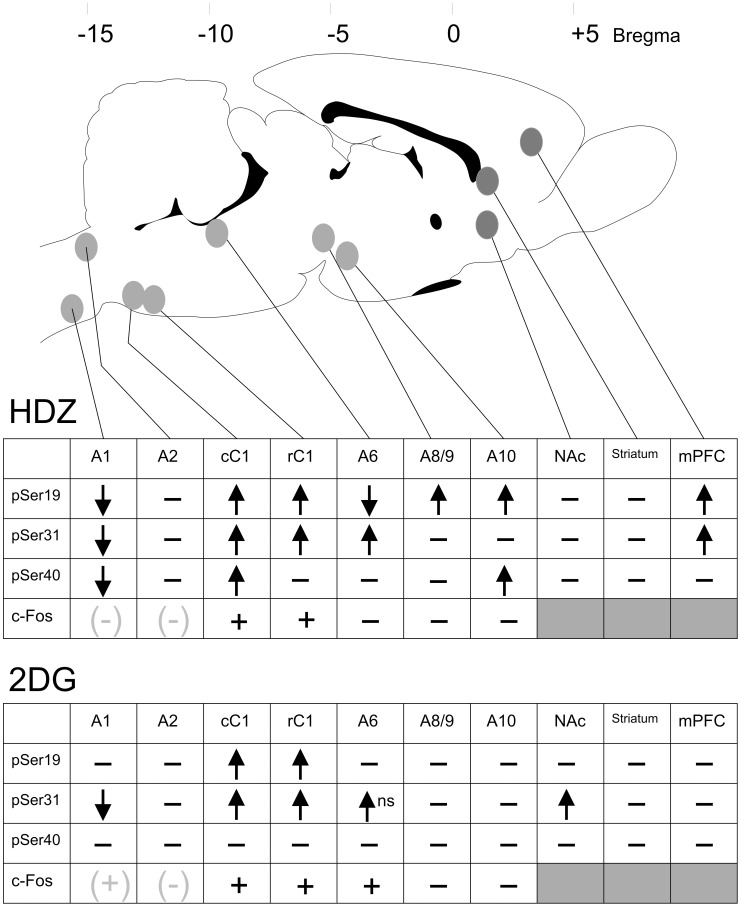
A summary of the changes in pSer19, pSer31 and pSer40 (30 min) and c-Fos expression (120 min) from 10 brain regions evoked by 2DG or HDZ. Decreases in phosphorylation are shown by downward arrows and increases by upward arrows. No changes are indicated by a horizontal dash. Increases in c-Fos are indicated by plus and no c-Fos expression is indicated by a horizontal dash. c-Fos data utilized from other studies is indicated in grey symbols [6], [34].

The directional changes in phosphorylation of the serine residues of TH and the presence or absence of c-Fos-ir in each brain region, in response to both stimuli after 30 min are summarized in Figure 11. 2DG never evoked a change in pSer40 whereas HDZ evoked a complex pattern of phosphorylation changes in multiple brain regions. The pattern of TH phosphorylation evoked did not always match the c-Fos expression.

### The Effect of HDZ on TH Activity in the Caudal C1 and Medial Prefrontal Cortex

**Figure 12:**
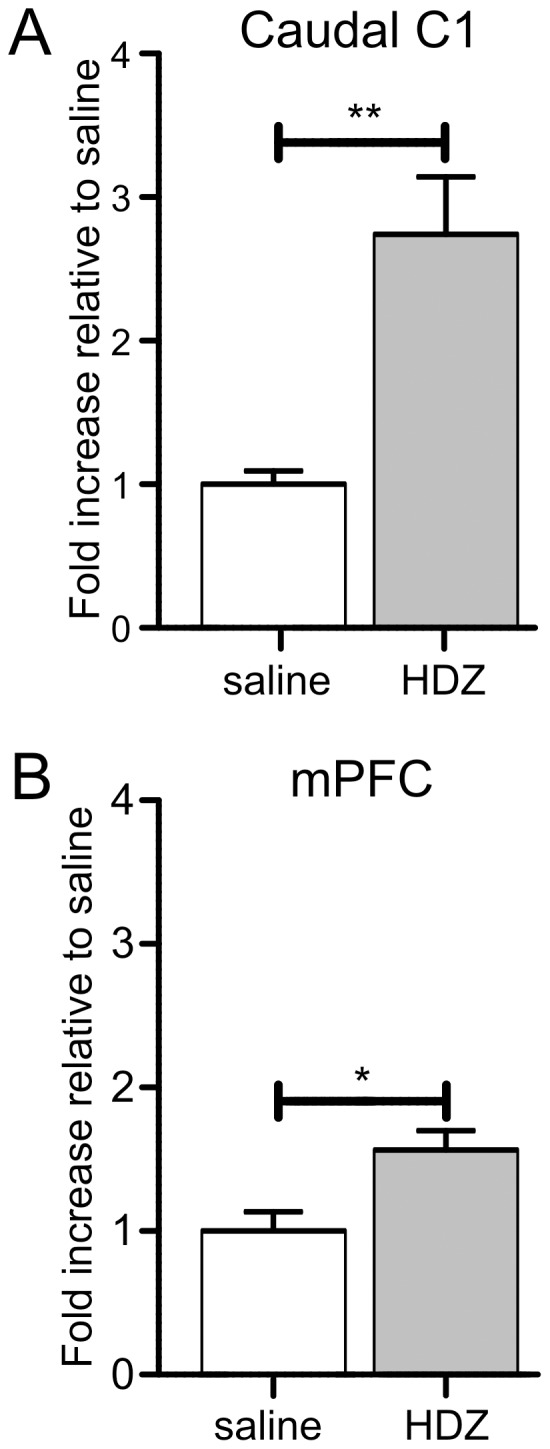
Changes in the activity of the enzyme, tyrosine hydroxylase (TH), 30 min following the administration of HDZ (10 **mg/kg i.p.) in the caudal C1 (n = 5) (A) and medial prefrontal cortex (mPFC, n = 5) B).** A) In caudal C1 a large increase in TH activity was seen when compared to saline treatment (P<0.01) whereas B) in the mPFC a smaller but significant increase in TH activity (P<0.05) was evoked.

It has been demonstrated that TH phosphorylation *in vitro* increases TH activity to restore catecholamine levels after catecholamine release [8]. We next determined the extent to which TH phosphorylation *in vivo* increases TH activity (Fig. 12). Two regions with distinctly different patterns of TH phosphorylation, caudal C1 and medial prefrontal cortex were examined 30 min following HDZ (n = 5 for each site) or saline (n = 5 for each site). Our prior observations established that HDZ caused a significant increase in TH phosphorylation at all three serine residues in the caudal C1 (Fig. 5C), and caused significant TH phosphorylation at Ser31 and Ser19, but no change in Ser40 in medial prefrontal cortex (Fig. 10C). TH activity determined by radioimmunoassay in the caudal C1 area was increased by 2.7 fold (p<0.01, Fig. 12A) and in the medial prefrontal cortex by 1.6 fold (p<0.05, Fig. 12B).

## Discussion

The major findings of the study are 1) TH phosphorylation changes were identified in discrete brain regions following hypotensive or glucoprivic stimuli, with some regions activated by both, some by only one and some by neither stimulus, 2) hypotension evoked phosphorylation changes were seen in multiple brain regions, whereas glucoprivic evoked effects were more restricted, 3) TH phosphorylation changes were identified in brain regions containing catecholaminergic cell bodies (and terminals) or terminals alone, 4) increases in TH phosphorylation reflected increases in TH activity in sites containing terminal fields, with or without cell bodies, 5) increases in neuronal activation as measured by c-Fos expression reflected changes in TH phosphorylation in some brain regions, however changes in TH phosphorylation were also a sensitive measure of activation/inhibition in some sites such as the midbrain dopaminergic cell regions that showed no c-Fos expression, 6) different patterns of serine residue phosphorylation were often evoked by the two stimuli in the same brain region, suggesting different levels of neuronal activation and/or cellular signaling, 7) TH phosphorylation at individual serine residues was seen to increase, decrease or not change, 8) the TH phosphorylation changes were, in general, transient with larger changes evident at 30 min compared to 60 min after the stimulus.

These findings indicate that changes in site specific TH phosphorylation can be used as a short term marker of neuronal activation at brain sites containing either catecholaminergic cell bodies and/or terminals. Furthermore, select catecholaminergic regions are activated by each acute stimulus and the catecholamine mediated consequences of hypotension are more common than those of glucoprivation. Catecholamines are most likely released or metabolized at some brain sites which contribute to counter regulatory responses evoked by each stimulus. Different TH phosphorylation patterns reflect intracellular signaling cascades evoked by diverse receptor mechanisms in different brain regions.

### Methodological Considerations

The dose and route of administration of each drug (stimulus) used was based on previous studies where maximal effects were evoked [3], [6], [17], [20], [24], [26] and the physiological responses evoked here are distinct for each stimulus (except they both induce a corticosterone response), and do not differ from those described previously. Plasma corticosterone was elevated indicating activation of the hypothalamic-pituitary-adrenal axis [27]–[28]. As both stimuli evoke increases in TH phosphorylation and c-Fos in caudal C1 and these neurons innervate hypothalamic neurons that contain corticotrophin releasing hormone, the caudal C1 group could be responsible for the observed increases in plasma corticosterone. In support of this, selective lesions of caudal C1 eliminated the corticosterone response evoked by glucoprivation [29]. Importantly, as similar levels of corticosterone were evoked by both stimuli this cannot be the primary cause of TH phosphorylation change in other brain regions, as these were distinct for each stimulus.

The time points chosen for analysis (20–30 and 50–60 min) were based on our previous studies [17] where the most significant changes in phosphorylated TH in the adrenal gland following 2DG administration were found to occur at these two time points. Furthermore, phosphorylation at Ser19 is usually considered the site to be phosphorylated most rapidly and robust increases were identified in several brain regions. It remains a possibility however that some changes in phosphorylation may have occurred more rapidly.

The phospho-specific TH antibodies used here have been generated, well characterized and tested against specific TH serine residues [18], [30]. No cross reactivity between antibodies was seen. Furthermore phospho-specific and non- phosphorylated recombinant TH were included in each immunoblot ensuring appropriate identification of each site and TH protein respectively. The antibodies were also tested using immunohistochemistry confirming there usefulness for selective labeling phophorylation of TH-ir neurons.

### Causes and Consequences of the Phosphorylation of Specific Serine Sites on TH

Ser19 phosphorylation was increased in the caudal C1, rostral C1, A8/9, A10 and medial prefrontal cortex following one or both stimuli. In adrenal cell cultures Ser19 is phosphorylated rapidly and then is dephosphorylated sometimes to below basal levels [8], [16], [31]. Phosphorylation of Ser19 was seen after 30 min but never at 60 min confirming the speed of the neuronal response and its transient nature at multiple brain sites *in vivo*. Phosphorylation at Ser19 depends primarily upon the activation of CaMKII in response to increases in levels of Ca^2+^ which enters the cell after depolarization [8]. This suggests that catecholaminergic neurons showing increased pSer19 have been depolarized by the stimulus. Phosphorylation of Ser19 alone does not change the activity of TH and so does not reflect a need for more catecholamine synthesis [8]. Dephosphorylation can result from a decrease in protein kinase activity in the continued presence of protein phosphatase activity, or to an activation of protein phosphatases, some of which are also calcium dependent [8], [32]–[33]. If calcium influx is rapid and substantial, dephosphorylation may override phosphorylation leading to a decrease in protein phosphorylation [32]. Dephosphorylation was evident in the A1 and A6 regions in the present study and we speculate that the decrease in Ser19 in A1 at least suggests a decrease in kinase activation as little evidence supports depolarization of this site by a normovolemic hypotensive stimulus [34].

Changes in pSer31TH were seen in the caudal C1, rostral C1, A6, the nucleus accumbens and the medial prefrontal cortex following one or both stimuli. In adrenal cell cultures Ser31 is slow to be phosphorylated and very slow to be dephosphorylated [16], [31]. In keeping with this and our other studies *in vivo* [17], [35] a change in Ser31 phosphorylation was evident at both 30 and 60 min after the stimulus. Phosphorylation of Ser31 depends upon MAPK [8], [16], although CDK5 may also play a direct or indirect (via activation of MEK1) role [8], [16], [36]–[37]. In a previous study we have demonstrated that pMAPK is significantly elevated in the rostral C1 and A6 30 min following HDZ [38] suggesting that the increased pSer31 in these regions may result from activation of MAPK. Phosphorylation of Ser31 increases TH activity [10, 39, 40)] which in turn increases catecholamine synthesis [40]. Large increases in Ser31 phosphorylation were seen here in the medial prefrontal cortex and TH activity was concomitantly increased indicating the need for catecholamine synthesis, presumably due to catecholamine release or metabolism following hypotension. Thus Ser31 phosphorylation could represent an important mechanism to increase TH activity *in vivo.* Salvatore et al (2009) [15] has suggested that phosphorylation of Ser31TH rather than Ser40 controls dopamine availability and this may be the case for the medial prefrontal cortex and possibly the A6 region following hypotension as in neither case was there a change in pSer40TH.

Phosphorylation at Ser40 was only found in caudal C1 and the A10 region. In adrenal cell cultures Ser40 was phosphorylated at times following the phosphorylation of Ser19 and prior to the phosphorylation of Ser31 and was not rapidly dephosphorylated [8], [16]. In keeping with this Ser 40 was phosphorylated at both 30 and 60 min in caudal C1 in the present study. An extensive array of kinases can phosphorylate Ser40 (most commonly PKA) and phosphorylation is always associated with increases in TH activity and catecholamine synthesis [8]. In line with this TH activity was increased in caudal C1 at 30 min, although Ser31, which can also contribute to TH activity, was phosphorylated.

In the present study phosphorylation at Ser31 was often evoked, as were changes in Ser19, with changes in Ser40 least commonly elicited. The ease with which Ser31 is activated in the brain does not appear to depend upon the stimulus used, the brain site activated [12], [15], [41] or the catecholamine produced.

### The Activity of Brain Regions in Response to the Stimuli

2DG evoked changes in TH phosphorylation in a restricted population of brain regions (4 of 10 examined) compared to HDZ (7 of 10 examined). On the other hand c-Fos-ir revealed neuronal activation following 2DG in only 3 regions (cC1, rC1 and A6) and HDZ in only 2 brain regions (cC1, rC1) when data from the present study and the data of others pertaining to the A1 and A2 regions is considered [6], [34]. The A2 region and dorsal striatum were unaffected by either stimulus suggesting that catecholamines in these regions are not involved in the counter regulatory responses evoked.

### Glucoprivation Evoked Changes in TH Phosphorylation

2DG evoked dephosphorylation in the A1 region and increased phosphorylation in the caudal C1, rostral C1 regions and the nucleus accumbens.

#### The A1 region

In the A1 region pSer31 was decreased in response to 2DG. This was surprising as c-Fos was detected in some A1 cells [6] and, DBH mRNA was increased [42]. As described above the reduced phosphorylation could indicate a high calcium entry rate although less half the A1 neurons were activated [6]. Any activation of A1 may be associated with the reported increase in vasopressin [43].

#### The rostral and caudal C1 regions

Increased pSer19TH was evident in caudal and rostral C1 regions in response to 2DG. This is in keeping with c-Fos activation of C1 cells seen previously [6] predominantly in the caudal C1 cells. Furthermore destruction of caudal C1 selectively reduced the hypothalamically mediated feeding response evoked by glucoprivation [20], [26]. Here we show that rostral C1 neurons are also activated. This finding is supported as some bulbospinal neurons with myelinated axons, indicative of rostral C1 neurons, are excited by systemic 2DG [44] and selective destruction of rostral C1 cells impairs the adrenal secretion of adrenaline evoked by 2DG [20], [26] which would normally act to increase glucose mobilization. Our data indicate that new catecholamine may be synthesized in the caudal C1 region in response to 2DG as pSer31 and pSer40 were increased within 60 min of the stimulus. This is the first evidence to suggest that catecholamines in the caudal C1 region play a role in response to glucoprivation. Whether or not catecholamine is synthesized in the rostral C1 region is less clear as only Ser31 is increased, although at both time points examined. However, it should be noted that the rostral C1 cells excited by 2DG were difficult to find [44] suggesting their paucity, perhaps reflecting the lower phosphorylation signal. It is unclear at this point whether catecholamines are released at the dendrites/soma of C1 cells following physiological stimuli (although there is some support for this idea [45]) or whether the newly synthesized catecholamine could be transported to distant terminals for release. It is unlikely that A5 [45] or A1 cell groups ([46] however see [45]) which provide catecholaminergic input to C1 cells could be the source of the TH phosphorylation as A5 cells are not activated following 2DG [6] and dephosphorylation was seen in A1. These data suggest for the first time that C1 cells are synthesizing new TH in order to replenish adrenaline stores released in response to glucoprivation.

#### The nucleus accumbens

2DG evoked phosphorylation of Ser31 in the nucleus accumbens which must arise from the TH containing terminals. This is in keeping with previous findings where hypoglycemia increased dopamine release in the region [47] and 2DG evoked c-Fos in neurons in the area [4] although, a reduced dopamine turnover [48] has also been reported. Our data also support the idea that Ser31 activity is related to dopamine turnover in the brain [15]. Nucleus accumbens receives input from A6, A8, A9 and A10 cell group [49]. As dopamine is released, inputs from A8, A9 and A10 are most likely and the increase in pSer31 must arise from cortically acting mechanisms as A8–10 do not express c-Fos or show phosphorylation changes in response to 2DG. TH phosphorylation in the nucleus accumbens evoked by glucoprivation may be related to its well described role in ingestive behaviour [50].

#### The A6 region

Although the increases pSer31TH in A6 evoked by 2DG did not reach significance about 60% of A6 neurons did contain c-Fos-ir. There have been inconsistent reports of c-Fos-ir in A6 following 2DG or hypoglycemia with only a proportion of the TH-ir cells activated, if at all [6], [51]. A higher dose of 2DG did cause an increase in TH mRNA [51] suggesting TH turnover may occur. Whether input arising from A10 or A6 cells themselves play any role in the response to 2DG is difficult to determine. It is possible that phosphorylation occurred earlier than the first time point measured here as immobilization evoked phosphorylation in A6 only at 10 min, but not later [35]. A role for the A6 region in glucose homeostasis is suggested by it connections with the suprachiasmatic nucleus [52].

### Hypotension Evoked Changes in TH Phosphorylation

In contrast to that seen following 2DG, a wide range of brain sites showed altered phosphorylation of TH in response to hypotension.

#### The A1 cell region

Decreases in pSer19, pSer31 and pSer40 TH were seen in the A1 cell group in response to hypotension. This may reflect a reduced activation as c-Fos levels were similar to control when a similar stimulus was used [34]. Although the activation of A1 is pivotal to hemorrhage evoked elevation in vasopressin [53] the possibility of reduced A1 activation in response to normovolemic hypotension has not previously been postulated.

#### The rostral and caudal C1 cell region

Caudal C1 shows the most robust pattern of increased phosphorylation seen in this study with all three serine residues phosphorylated in response to hypotension. Rostral C1 is also phosphorylated at both Ser19 and Ser31. Hypotension evoked c-Fos-ir in caudal and rostral C1 as demonstrated here and elsewhere [24], [34], [54] in keeping with the fact that all C1 neurons are highly barosensitive [55]. Our data show that TH activity is increased in caudal C1, and most likely in rostral C1, indicating that catecholamine is released. This is confirmed by previous findings in which adrenaline was released in the rostral C1 region [56] and catechol current was increased in the caudal C1 [57] and rostral C1 regions [58] by hypotension. These data validate the finding that TH phosphorylation leading to increased TH activity correlates with catecholamine release *in vivo*. The potential sources of adrenaline release are local C1 cells or contralateral C1 [59]. Caudal C1 but not rostral C1 cells express somatic VMAT2-ir [60] and local unilateral injection of anti-DBH conjugated to the toxin, saporin, kills C1 cells both ipsi- and contralaterally [45], supporting the ideas of somatodendritic release/uptake of adrenaline and a contralateral C1 projection. The HDZ evoked TH-phosphorylation is likely to be mediated by baroreceptor unloading as both adrenaline release and c-Fos activation in C1 neurons are inhibited by sino-aortic denervation [24], [56]. The rostral C1 neurons serve to maintain vasomotor tone and release noradrenaline from the adrenal medulla and caudal C1 is potentially involved in increasing vasopressin secretion, all in order to counter the hypotension. The role that local catecholamine release in the C1 region plays has yet to be determined as the increase in vasomotor tone mediated by rostral C1 is mediated at the level of the spinal cord by glutamate.

#### The A6 cell region

The noradrenergic A6 region shows an increase in pSer31 but a decrease in pSer19 that could indicate kinase inhibition/phosphatase excitation supporting the finding of little to no c-Fos activation. Previous studies show mixed levels of c-Fos activation following hypotension [6], [20], [54], [61]–[63]. Alternatively excessive calcium entry indicative of depolarization could lead to the phosphorylation patterns seen which is in keeping with increased activity of activity of A6 neurons during hypotension [64] and the release of noradrenaline in the region 15 min and DOPAC 45 min following the hypotensive stimulus [21] [65]. However the A6 cell group receives adrenergic innervation from C1 [66]–[68] as well as dopaminergic input from A10 [69] thus it remains a possibility that other catecholamines may be released in the A6 region in response to hypotension. Most commonly the A6 is associated with suppression of the baroreflex [70].

#### Dopaminergic midbrain regions A8/9 and A10

An increase in TH phosphorylation at Ser19 in A8/9 and at Ser19 in A10 indicates these regions are activated and the increases in Ser 40 in A10 suggest that TH activity is increased, in response to hypotension. It is possible that the increases in pSer19 seen in the A8/9 region may result from contamination by the closely adjacent A10 cells. As chemical activation of the A10 region evokes cardiovascular responses and decreases in baroreflex sensitivity [71] a role in the response to hypotension may not be unexpected [72]–[74]. It is curious however that following modest hypotension catechol current was not detected at A10 however more severe hypotension was not tested even though in the same study in the C1 region this stimulus evoked the largest catechol currents [58]. The source of phosphorylated TH could be the dopaminergic cells themselves which exhibit somatodendritic dopamine release or the noradrenergic inputs arising from A6 [49], [69].

In contrast to other sites in the present study changes in pSer19 did not correlate with c-Fos induction in the A8/9 or A10 regions nor do previous studies indicate that c-Fos is activated in these regions following hypotension. It has been suggested that c-Fos may not be easily induced in midbrain dopaminergic neurons [75]. We however show clear evidence of TH phosphorylation following hypotension suggesting that this may be a powerful tool to demonstrate neuronal activation in the midbrain dopaminergic cell groups. No changes in pSer31 were evoked in these areas indicating the lack of involvement of MAPK or CDK5 perhaps in keeping with the strong correlation between pMAPK and c-Fos [76]. It is possible however that catecholamine release serves to inhibit cells in the region.

#### The medial prefrontal cortex (mPFC)

Hypotension evoked increases in both pSer31TH and TH activity in the mPFC suggesting catecholamine release. This must arise from terminal fields of the A6 and/or A10 cell groups [69] and previous data show that both noradrenaline and dopamine are released in the mPFC following hypotension [21], [77]. This further validates the notion that increased pSer31 without Ser40 phosphorylation increases TH activity *in vivo* and reflects catecholamine release. Baroreceptor denervation reduces c-Fos activation in higher brain regions [2] but as activation was not seen previously in A8/9 or A10 or in the mPFC barodenervation studies are now required.

### Conclusions

Thus, this study demonstrates that the TH phosphorylation profile is a powerful and reliable marker for acute activation in catecholamine containing brain regions including dopaminergic cell groups and regions containing only terminal fields. This has advantages over other methodologies, such as c-Fos, as changes can be detected within 20–30 min of a stimulus, time course data can be determined, both increases and decreases in phosphorylation can be detected and phosphorylation in nerve terminals can be identified. Most importantly, the action measured is of a single neurochemical entity providing chemical specificity. Some evidence for anatomical resolution is provided by immunohistochemical appraisal of the pSerTH as demonstrated here however difficulties associated with quantitative analysis of such data together with small ranges of minimal to maximal stoichiometry may reduce the impact of using immunohistochemical data in isolation. Although other phosphorylated markers including pMAPK and pCREB can be very useful [38] for identifying activated neurons/pathways early following a stimulus these do not necessarily inform about the neurotransmitter/s utilized. The immunohistochemical expression of pCREB/pERK may reflect changes other than in the phosphorylation of TH (even though CRE is a critical controller for the transcription of TH) even if identified in a TH containing neuron. Furthermore changes in pCREB and pMAPK may also reflect changes in the activity of glia at least if used for Western blotting. Similarly monitoring primary (heteronuclear) TH transcripts using intronic probes could be used to provide cellular localisation of changes in TH however they monitor changes over a different timecourse to phosphorylation, as they have been initially detected at 6 hours post stimulus [78]. Maximum information will be gained using a combination of these techniques to reveal neural pathways as well as chemical messengers and their signaling cascades that are recruited in response to different stressors.

The identification of changes at three serine residues on TH provides information not only regarding neuronal activation (Ser19) but also has the potential to indicate signaling pathways activated (e.g. MAPK or CDK for Ser31) and the probability of catecholamine synthesis/metabolism being activated (if Ser31 and particularly Ser40 are phosphorylated). Ser 19 and Ser31 are the sites most sensitive to phosphorylation in the brain regions examined and although phosphorylation of Ser31 may indicate dopamine turnover at higher brain regions [15] it appears to reflect the actions of other catecholamines in the brainstem.

Different patterns of phosphorylation were detected in the same cell group by different stimuli indicating that different levels of activation or perhaps different cell signaling mechanisms are recruited. However some changes detected included directionally opposite effects at different serine residues in the same cell group. This complex pattern raises a challenge to determine what is happening at the cellular level. This not only involves monitoring the three serine residues that demonstrate changes in the activity of the enzyme but also indicates the importance of monitoring the time course of these changes. In the present study only two time points were measured based on previous findings in the adrenal medulla utilizing similar stimuli. Although significant changes were detected at 20–30 min, the earliest time point investigated here it remains a possibility that some phosphorylation changes may be detected earlier.

Our findings demonstrate that central responses to glucoprivation and hypotension rely not just on neural pathways containing catecholamine but on the involvement of catecholamines themselves, at least in some brain regions. In future studies it will be important to determine the prevalence of somatodendritic release of catecholamines in the brainstem in response to these stimuli and also to determine in higher brain regions particularly A6, A10 and medial prefrontal cortex that hypotension evoked changes are baroreceptor mediated.

## Materials and Methods

### Reagents

Hydralazine (HDZ), 2-Deoxy-D-glucose (2DG), ethylene glycol tetraacetic acid (EGTA), ethylene diamine tetraacetic acid (EDTA), Tween-20, reduced glutathione and mouse anti-tyrosine hydroxylase antibody (catalogue # T1299) were purchased from Sigma Chemical Co. (St Louis, MO, USA). Criterion Tris-HCl precast gels (10%) and molecular-weight PAGE standards were from Bio-Rad Laboratories (Hercules, CA, USA). Nitrocellulose membrane (Hybond ECL) and ECL Advanced kit were from GE Health Care (Little Chalfont, UK). Anti-rabbit immunoglobulin (horseradish peroxidase-linked whole antibody from goat, catalogue # 111-035-144), anti-mouse immunoglobulin (horseradish peroxidase-linked whole antibody from goat, catalogue # 115-635-035), Cy3 donkey anti rabbit IgG (catalogue # 711-166-152) and DyLight 488 AffiniPure Donkey Anti-Mouse IgG (catalogue # 715-485-150) were from Jackson Immunoresearch Laboratories Inc. (West Grove, PA, USA). Anti-sheep immunoglobulin (horseradish peroxidase-linked whole antibody from rabbit, catalogue # 31480) was from Pierce Biotechnology (Rockford, USA). Rabbit anti-c c-Fos antibody was from Santa Cruz Biotechnology Inc**.** (Santa Cruz, CA, USA, catalogue # sc-52). Phospho-Ser40TH, phospho-Ser31TH, and phospho-Ser19TH specific antibodies were generated and tested for specificity as described by [18], [30].

### Ethics and Animals

All experiments were carried out with the approval of the Animal Ethics Committees of Macquarie University, Sydney and conducted in accordance with the Australian Code of Practice for the Care and Use of animals for scientific purposes.

Animals (male Sprague-Dawley (SD) rats (300–500g, 10–13 weeks of age)) were purchased from the Animal Resource Centre (Perth, Australia). One day prior to experimentation rats were housed singly in a temperature controlled room (21±1°C) with ad libitum access to food and water. Multiple cages were housed in a single room for a minimum of 12 hours and rats were always within sight, sound and smell of other rats in order to reduce the stress of isolation.

### Heart Rate and Blood Pressure Measurement

Rats that were anesthetized with 10% urethane (1.3 g kg^−1^ i.p.) and ventilated with oxygen during the procedure. The left femoral vein and artery were cannulated for drug administration and AP recording. Supplemental doses of urethane were administered i.v. if required. Core temperature was measured with a rectal probe and maintained at 37°C with a homeothermic blanket and an infra-red lamp. After a stable recording of AP and heart rate was achieved, 2DG (400 mg/kg i.v. n = 3) or HDZ (10 mg/kg i.v. n = 3) was administered.

### Protocol for Western Blot Analysis

In conscious animals HDZ (10 mg/kg, n = 10), 2DG (400 mg/kg, n = 10) or saline (vehicle, n = 10), was administered intraperitoneally (0.4 ml) and food and water were removed. Animals were sacrificed 20–30 min (called 30 min) or 50–60 min (called 60 min) following treatment by an anaesthetic overdose, sodium pentobarbital (Lethabarb, 80 mg/kg i.p.). When rats no longer responded to painful stimuli they were decapitated by guillotine (<5 min) and blood and tissue samples were collected.

### Blood and Tissue Collection and Analysis

Whole trunk blood (5 ml) was collected into tubes containing 4 mM EGTA and 4 mM reduced glutathione as described previously (Lambert et al. 1995). Blood samples were centrifuged for 10 min at 1800 rpm (4°C). Resulting plasma was then spun for 10 min at 2700 rpm (4°C) and then stored at −80°C until analysis. Plasma corticosterone level was measured using an ^125^I Corticosterone RIA kit (MP Biomedicals, Orangeburg, NY) according to the manufacturer’s instructions. Blood glucose was measured immediately using Accu-check performa glucometer from Roche (Mannheim, Germany).

The brain was rapidly removed from the skull and placed into ice-cold buffer before being placed into a plastic brain matrix (World Precision Instruments, USA). One to 3 mm thick coronal sections were isolated containing the 10 areas of interest based on the rat brain atlas [79]. The areas of interest were rapidly dissected and isolated brain regions refrozen on dry ice and kept at −80°C until analysis. The areas of interest ranged in size from ∼1- ∼2.5 mm^3^. All sites except the VTA and SN were taken from sections 1 mm thick which were taken from the same 3 mm thick section.

#### Brain regions of interest

Ten brain regions containing catecholaminergic cell bodies and/or terminals were isolated by microdissection and their dorsoventral and mediolateral dimensions are illustrated in Figs 4,5,6,7,8,9,10. The regions included that containing the A1 cell group (Bregma -14.0 to-15.0 mm), the A2 cell group (Bregma −14.0 to −15.0 mm), the caudal C1 cell group (Bregma −13.0 to −14.0 mm), the rostral C1 cell group (Bregma −12.0 to −13.0 mm), the A6 cell group (Bregma −9.0 to −10.0 mm), the ventral tegmental area (VTA) also including the central linear nucleus (Bregma −4.0 to −7.0 mm), the substantia nigra (Bregma −4.0 to −7.0 mm), the nucleus accumbens and the dorsal striatum (Bregma +1.0 to+2.0 mm), and the medial prefrontal cortex (Bregma +2.5 to +3.5 mm).

### Measurement of Site-specific TH Phosphorylation and TH Protein in Specific Brain Area

Brain regions were weighed and homogenised, in 20–40 volumes of homogenization buffer (2% SDS, 2 mM EDTA, 50 mM Tris (pH 6.8)), by sonication, on ice (3 times×30 sec at 10,000 A), then boiled for 5 min and centrifuged for 20 min. Supernatants were carefully removed. 100 µl of supernatant was mixed with 5 µl of 10% dithiothreitol (DTT) and 35 µl of sample buffer (40% glycerol, 50 mM Tris, minimal bromophenol blue, pH 6.8). 25 µL of each sample was run on Criterion Tris-HCl precast gels (10%) and then transferred to nitrocellulose as described previously [31]. Additional lanes of every blot contained recombinant rat TH (rTH) specifically phosphorylated to full stoichiometry at Ser40 (by PKA), or Ser31 (by MAPK1), or Ser19 (mutated form of rat TH (Ser40Ala) phosphorylated by CaMKII-to avoid concomitant Ser40 phosphorylation) and also non-phosphorylated recombinant rat TH. Membranes were immunoblotted with pan or phospho-specific TH antibodies overnight at 4°C. The levels of pSer19TH, pSer31TH, pSer40TH and total TH (tTH) protein were determined using specific antibodies that have previously been characterized [18], [30] where it was shown that the phospho-specific antibodies did not significantly cross-react with non-phosphorylated rTH, or with rTH phosphorylated at the alternative Ser residues. Secondary antibodies (goat anti-rabbit or rabbit anti-sheep immunoglobulin) were applied to the membranes for 1 hr at room temperature. The immunoblots were visualized and quantified on the Fuji LAS 4000 imaging system (GE Health Care, Little Chalfont, UK) using ECL-advanced detection reagents. The density of the bands was measured by selecting the specific area of the protein band and the subtracting the background. All analysis were done using Fujifilm Multigauge v3.0 software (Tokyo, Japan) and expressed as a fold increase relative to the control samples (animals treated with saline). Site-specific TH phosphorylation was expressed as the ratio of TH phosphorylation at Ser19, Ser31 or Ser40 to total TH protein, to account for variability in total TH protein between samples.

### TH Activity Assay

The caudal C1 (n = 5 for saline and HDZ) and mPFC (n = 5 for saline and HDZ) were homogenised in homogenization buffer (60 µl, 2 mM Kphos buffer pH7.4, 1 mM EGTA pH7.5, 1×protease inhibitor cocktail tablet, 1 mM DTT, 80 µM ammonium molybate, 1 mM sodium pyrophosphate 1 mM, 1 mM sodium vanadate, 5 mM β-glycerolphosphate and 2 µM microcystin). Samples were sonicated 3 times×30 seconds at 4°C, using a micro-sonicator, and then centrifuged for 20 min at 16,000 rpm. Supernatants were collected and protein levels were determined using a BCA protein assay kit (Thermo Fisher Scientific, IL, USA) according to the manufacturer’s instructions. TH activity was measured using a method based on the tritiated water release assay with minor modifications [80]. The reaction mixture contained 50 µg sample, 36 µg catalase, 2 mM potassium phosphate (pH 7–7.4), 0.008% β-mercaptoethanol, 24 µM tyrosine, 1 µCi 3,5-[^3^H] tyrosine and 100 µM tetrahydrobiopterin, final volume 50 µl. Assays were performed for 20 min at 30°C and were stopped by addition of 700 µl charcoal slurry (7.5% activated charcoal in 1 M HCl). Unbound ^3^H_2_O was analysed by scintillation spectrometry (Wallac1410, Pharmacia, Turko, Finland) for 20 min per sample. The changes in TH activities were expressed as a fold increase of the mean of the basal samples.

### c-Fos Protein and pSerTH Immunohistochemistry and Analysis

To detect c-Fos protein animals were administered HDZ (10 mg/kg i.p., n = 3), 2DG (400 mg/kg i.p., n = 3) or saline (n = 3) and left for 2 hours in a quiet environment and deprived of food and water. To detect phospho-TH animals were administered HDZ (10 mg/kg i.p., n = 3) or saline (n = 2) and left for 30 min. All animals were anesthetized with sodium pentobarbital (Lethabarb, 80 mg/kg i.p.) and perfused trans-cardially with 300 ml ice-cold saline followed by 400 ml ice cold fixative solution (4% PFA/0.1 M PB, pH 7.4). The brain was removed rapidly and stored in the same fixative (12–16 hrs, 4°C). The following day, the brain was blocked and serial sections (40 µm thick, 200 µm apart) were cut on a vibrating microtome (Leica VT 1000S, Wetzlar, Germany) into ice-cold 0.1 M PBS. After three 30 min washes in 0.01 M tris phosphate buffered saline (TPBS), sections were incubated in a solution containing 1∶4000 mouse anti-tyrosine hydroxylase antibody and 1∶1000 rabbit anti-c-Fos antibody with 5% normal horse serum in TPBSm or sections were incubated in a solution containing 1∶1000 of rabbit anti- pSer19TH or 1∶2000 rabbit anti-pSer31TH antibody, for 48 hrs at 4°C. After washing three times for 30 min each in TPBS, sections were then incubated overnight at 4°C in a solution containing 1∶500 Cy3 donkey anti rabbit IgG or together with 1∶500 DyLight 488 Donkey Anti-Mouse IgG with 2% NHS in TPBSm. The next day sections were washed 3 times for 30 min, and mounted onto glass slides and coverslipped with ProLong Gold (Invitrogen, CA, USA).

For analysis of c-Fos and TH immunoreactivity anatomically matched sections for each brain region were analyzed for all animals in each treatment group (HDZ, 2-DG or saline). Optical sections of each region were taken using a Zeiss Z2 microscope (Zeiss, Germany) at 2 µm intervals (in the z direction) using filters selective for each fluorophore. Images were then analyzed and the number of cells showing immunoreactivity for TH were counted and assessed for immunoreactivity for c-Fos. For phospho-specific TH immunoreactivity, anatomically matched sections were photographed. Further analysis was not attempted as immunohistochemistry cannot be reliably used for quantitative assessment.

### Statistical Analyses

Statistical analyses were performed using Student’s unpaired t-tests for single comparisons. Differences were considered to be significant at the p<0.05 level.
